# Economic evaluation: costing participatory learning and action cycles with women’s groups to improve feeding, care and dental hygiene for South Asian infants in London

**DOI:** 10.3389/fpubh.2025.1601990

**Published:** 2025-10-13

**Authors:** Yeqing Zhang, Priyanka Patil, Shweta Chaudhary, Kunal D. Kondhare, Darlington David Faijue, Monica Lakhanpaul, Michelle Heys, Joanna Drazdzewska, Clare H. Llewellyn, Kelley Webb-Martin, Carol Irish, Mfon Archibong, Jenny Gilmour, Phoebe Kalungi, Jolene Skordis, Logan Manikam, Neha Batura, the NEON Steering Team, Atul Singhal

**Affiliations:** ^1^Institute for Global Health, University College London, London, United Kingdom; ^2^Department of Epidemiology and Public Health, Institute of Epidemiology and Health Care, University College London, London, United Kingdom; ^3^Aceso Global Health Consultants Pte Limited, Singapore, Singapore; ^4^The Migrant Health Research Group, Institute for Infection and Immunity, City St. George’s University of London, London, United Kingdom; ^5^Nottingham University NHS Trust, Nottingham, United Kingdom; ^6^UCL GOS Institute of Child Health, University College London UCL, London, United Kingdom; ^7^Collaborative Centre for Inclusion Health, London, United Kingdom; ^8^Specialist Children’s and Young People’s Services, East London NHS Foundation Trust, London, United Kingdom; ^9^Women and Children First, London, United Kingdom; ^10^Department of Behavioural Science and Health, University College London, London, United Kingdom; ^11^Children’s Health 0-19 Service, London Borough of Newham, London, United Kingdom; ^12^Tower Hamlets GP Care Group, Mile End Hospital, London, United Kingdom; ^13^Global Business School for Health, University College London, London, United Kingdom

**Keywords:** participatory learning and action women’s groups, early childhood development, costs, affordability, nutritional intervention

## Abstract

**Background:**

The Nurture Early for Optimal Nutrition (NEON) programme was designed to promote equitable early childhood development by educating mothers of South Asian origin in two boroughs (Newham and Tower Hamlets) in East London on optimal feeding, care, and dental hygiene practices. The study found that the adapted Participatory Learning and Action (PLA) approach was highly acceptable and well-received by participants, with improvements in maternal confidence, infant feeding practices, and community engagement. However, gaps in specific feeding skills and challenges such as low attendance and retention rates were noted, particularly during the COVID-19 pandemic. This study conducted a cost analysis of the NEON programme and evaluated its financial sustainability.

**Methods:**

We conducted a financial and economic costing from the provider perspective, applying a stepdown procedure to identify costs associated with the development and implementation of the NEON programme. Estimates of total and average costs per mother are presented along with affordability assessments, expressed as a proportion of the borough’s annual child development expenditure. All costs were discounted and reported in 2022 pound sterling and in 2022 international dollars.

**Results:**

The total cost of NEON design and delivery was £68,165 ($INT 102,658), and the average cost per mother participating in the programme was £439($INT 661) in the face-to face arm and £407($INT 614) in the online arm. The largest contributor to the total cost was materials (50%), including NEON training manuals and intervention toolkits, vouchers for the community facilitators, and overheads, followed by staff costs (45%) and capital investments (5%). The total cost of intervention delivery in Newham accounted for around 0.047% of the borough’s annual child development expenditure, while the total intervention cost in Tower Hamlets was equivalent to 0.003% of its spending on children’s development.

**Conclusion:**

The delivery of NEON is largely within local authorities’ budget for childhood development. The unit cost is expected to decrease when sharing costs are spread across more participants and implementing systems are validated and well developed.

## Highlights

- This paper performs a cost analysis to understand the cost implications of running a participatory learning and actions intervention in London.
- This approach allows the incorporation of the local context such as the culture, beliefs, and existing practices, potentially leading to boosted accessibility and acceptability.
- This study deems that the NEON programme on evaluation is a financially sustainable model within the target population.

## 1 Introduction

Each year, over 240 million children under age 5 worldwide, face significant biological and psychosocial hazards that compromise their developmental potential (1). These hazards include malnutrition, exposure to violence and heavy metal, and inadequate cognitive and social-emotional stimulation (7). The first 5 years of life are crucial for brain development and the formation of caregiver-child attachments, therefore sensitive to early experiences (3). Exposure to adversity during this period has long-term negative impacts on physical and psychosocial health, as well as educational and economic achievements in adulthood (3–7). In monetary terms, the average income loss for adversely impacted children is estimated at 26% per annum (8). Additionally, there are intergenerational consequences as the developmental deficit and income loss perpetuates a cycle of poverty (8). At the same time, intervention during this early period has been shown to yield the greatest benefit for health and development (37). The benefit-cost ratios of such interventions have been estimated as approximately 18:1 for stunting reduction, 4:1 for preschool education, and 3:1 for home visits for underdeveloped children (8). The potential societal benefits far outweigh the costs, making such interventions particularly relevant in high- burden and resource-constrained settings.

While the majority of children at risk are concentrated in low- and middle-income countries (LMICs), these hazards may be generated or exacerbated by socio-cultural practices and economic constraints regardless of the geographic location (4). South Asia and Sub-Saharan Africa have the highest proportion of children under 5 at risk, at approximately 53 and 66%, respectively, (1). These statistics, along with similar estimates published in 2007 (4) directed a large number of early childhood interventions to these contexts (9). However, minority groups facing similar socio-cultural norms and economic disadvantages in high-income countries have largely been overlooked to date (10).

There is evidence to suggest that a large proportion of the minority ethnic population in the UK, especially those with South Asian heritage, experience significant social and economic disadvantage (11, 12). This may contribute in turn to poorer health outcomes. Children from South Asian families resident in the UK, are susceptible to many of the same developmental risks as young children resident in South Asia, including poorer birth outcomes and nutrition status (13, 14). The causes of these risks in the UK context are complex, including socioeconomic deprivation, discrimination, language barriers, cultural norms, and limited access to health information, among other factors (15). Early childhood interventions tailored specifically to these minority ethnic groups can improve health outcomes in early childhood and may help avert lifelong disparities in health, education, and economic outcomes. Unfortunately, the availability of such interventions is limited and there is a need for additional testing of targeted and effective interventions that can be delivered at low cost (16, 17).

The Nurture Early for Optimal Nutrition (NEON) programme, therefore, was designed to promote equitable early childhood development by educating mothers of South Asian origin in East London on optimal feeding, care, and dental hygiene practices (10). The intervention was delivered via participatory learning and action (PLA) cycles with women’s groups, which involved active participation, learning, and action by community members and mothers to identify and address problems together. Similar interventions in LMICs (18), including Bangladesh (19), Pakistan (20), and India (21), have been shown to be effective and cost-effective in reducing maternal and neonatal mortality, improving infant feeding, hygiene, and care practices, finally resulting in enhanced cognitive, language, and motor development outcomes among children participants (22). However, there is limited evidence of their success in disadvantaged areas of high-income countries. Adapting this approach for implementation in South Asian communities in East London, the NEON programme added evidence to the effectiveness and feasibility of PLA in high-income settings. This study conducted a cost analysis of the NEON intervention.

## 2 Methods

### 2.1 Study setting and intervention design

The study was conducted in two East London boroughs, Tower Hamlets (TH) and Newham (NH), both of which have large South Asian populations and experience high levels of socio-economic deprivation. According to the most recent census data, these areas exhibit higher levels of unemployment, lower average incomes, and poorer housing conditions compared to the rest of London (10). Furthermore, health indicators such as infant mortality rates, childhood obesity rates, and low breastfeeding rates are significantly higher in these boroughs compared to the London average (23). Early childhood health indicators, including immunisation uptake and access to nutritional support, are also lower in TH and NH, highlighting the need for targeted public health interventions in these communities (5). These socio-economic and health disparities make TH and NH particularly relevant for studying the impact of early childhood interventions.

The NEON pilot feasibility randomised controlled trial was run from December 2019 to May 2023 to assess the feasibility of a definitive trial (10, 24). A total of 12 clusters, i.e., borough wards, were equally randomised to an online treated arm, a face-to-face treated arm, and a control arm, balancing participant contamination while ensuring optimal representation of the South Asian population in East London, including Indian, Pakistani, Sri Lankan, and Bangladeshi communities (25). To enhance the accessibility and feasibility of the programme, Community Facilitators (CFs) from these ethnic backgrounds, as well as Health Visitors (HVs), General Practitioners (GPs), and midwives at each study ward, were recruited and involved in the development and delivery of the intervention. Recruitment of participants began in May 2022, and implementation started in September 2022; 263 mothers of infants under 24 months from TH and NH were enrolled. Participants in the treated arms received one PLA session every 2 weeks for a duration of 14 weeks and were followed up for 6 months afterwards. They were all provided with an intervention toolkit (24), including:

1. Picture cards detailing recommended feeding, care, and dental hygiene practices,
2. Healthy infant cultural recipes,
3. Participatory Community Asset Maps, and
4. A list of resources and services supporting infant feeding, care, and dental hygiene practices.

The control group received the standard care under the Healthy Child Programme 0–5, commissioned to local authorities, including regular mandatory postnatal visits and optional prenatal visits (10, 26). Single blinding was implemented for participant recruitment and outcome assessment. For detailed information on the study design, intervention packages, and implementation, please refer to the published NEON protocol (10).

### 2.2 Costing method

We conducted a financial and economic costing from the provider perspective. Cost data were sourced from the expenditure and accounting records of the implementing institutions in a trial-specific data collection Excel-based tool (10). We followed a stepdown procedure (2) to first identify all the costs incurred from the initiation of the intervention development to the completion of evaluation and dissemination. We distinguished between costs associated with start-up and implementation and excluded research costs. Costs associated with recruiting and monitoring the control group were also excluded. The monitoring and evaluation costs were too intricately linked to be separated from the implementation costs. Therefore, they were included as part of the implementation costs, as they are essential for successful programme delivery (27).

Costs were categorised into capital costs and recurrent costs. Capital costs included all goods and services having a useful life of more than 1 year, primarily computers and cameras. Their costs were recorded at their original purchase prices and depreciated at a rate of 20% per year to account for the value consumed during the intervention (28). Recurrent costs consisted of staff costs and materials. Staff costs included salaries for the study team who were responsible for designing and implementing the NEON intervention, which included activities such as coordinating local HVs, GPs, and Midwifery teams, and recruiting participants and CFs. They were valued based on the actual pre-tax salaries remitted. Other goods and services were deemed as materials, including PLA group facilitator manuals, intervention toolkits, rented venues and snacks for PLA meetings, staff training courses, vouchers for CFs, and overheads. These were valued at the original purchasing value. All costs were inflated or deflated to 2022 values. To improve evidence interpretability, discounting beyond inflation adjustment was not applied, as doing so would require further assumptions about the opportunity cost of funds. Results were reported both in 2022 pound sterling (£) and in 2022 international dollars ($INT).

### 2.3 Base case analysis

We evaluated the efficiency of service delivery by estimating two unit costs - the total cost per beneficiary and the implementation cost per beneficiary for both online and face-to-face arm. The total cost per beneficiary, (i.e., the total cost per mother) was calculated as the total cost divided by the number of participating mothers. The implementation cost per beneficiary (mother) was computed as the total cost per mother excluding fixed start-up costs in order to reflect the marginal cost of recruiting and treating one more participant. We conducted affordability analysis by calculating the total cost of NEON delivery as a percentage of the local authorities’ budget, and report this separately for Newham and Tower Hamlet.

### 2.4 Sensitivity analysis

We explored variation in service delivery efficiency by performing deterministic one-way sensitivity analysis on the total cost per mother. The base case reflected the best approximation of expected unit cost. Changes in assumptions and parameters, such as the number of effectively treated participants, the appropriate capital cost depreciation rate and the joint cost allocation, were individually assessed against the base case to gauge their influence on service efficiency. Firstly, we used the number of mothers who proceeded to the first PLA meeting as the denominator in the base case. We explored the unit cost variation by using the number of mothers who completed most PLA sessions, reflecting attrition. We also explored using the number of mothers who completed all six PLA meetings and were successfully followed up until the end of the study, under the stricter assumption of only those women being effectively treated. Second, we increased and decreased the capital good depreciation rate by 10 percentage points to account for the different degree of wear and tear of equipment. Third, we introduced variations to the joint cost allocation, varying the proportion of shared staff, capital, and other recurring costs assigned to PLA implementation and other undertakings like monitoring, evaluation, or research. By adjusting the initial allocation up and down by 20 percentage points, we derived a range of unit costs. This adjustment can reflect the shifting importance of various activities in future scale-up trials and replications in different contexts. The former scenarios may require increased monitoring and evaluation efforts, while the latter should put a greater emphasis on research.

## 3 Results

### 3.1 Base case

Since NEON was intended to be delivered as a programme in addition to usual practice or care, all the costs incurred were considered incremental costs. The total incremental cost of delivering NEON (Table 1) in two boroughs in East London was £68,165 ($INT 102,658). In Tower Hamlets, the total cost was £14,774 ($INT 22,249) and in Newham, £53,391 ($INT 80,409). In total, the start-up cost of £38,525 ($INT 58,021) accounted for 57% of the total cost, while the implementation cost of 29,639 ($INT 44,637) accounted for the rest of 43% (Table 1- Panel 2). Detailed results of total cost are presented in Table 1.

**Table 1:** Programme costs by component.

Cost component	Amount (2022 £)	Amount (2022 $INT)	% of Total cost
Panel 1:			
Total cost:	**68,165**	**102,658**	
Staff costs	30,622	46,117	45%
Materials	34,067	51,307	50%
Capital costs	3,476	5,235	5%
Panel 2:			
Cost by stage:			
Start-up	**38,525**	**58,021**	**57%**
Staff costs	11,875	17,884	17%
Materials	25,942	39,070	38%
Capital costs	709	1,067	1%
Implementation	**29,639**	**44,637**	**43%**
Staff costs	18,747	28,233	28%
Materials	8,125	12,237	12%
Capital costs	2,766	4,167	4%
Panel 3:			
Cost by arm:			
Face to face	24,158	36,382	55 women allocated, 6 PLA sessions
Online	44,007	66,276	108 women allocated, 8 PLA sessions
Panel 4:			
Cost by site:			
Tower Hamlets	14,774	22,249	57 women recruited
Newham	53,391	80,409	206 women recruited

Bold values indicate the overall cost, representing the sum of all subcategory costs listed below.

Materials’ costs contributed to half (50%) of the total costs, followed by staff costs (45%). The capital costs were minimal (5%) in comparison, and were mainly attributed to the electronic equipment required for advocacy, monitoring, and evaluation (Table 1-Panel 1). The costs incurred in these different categories during the start-up and implementation periods are presented in more detail in Table 1- panel 2.

Of the 263 women recruited, 186 met the inclusion criteria and provided consent to participate. Among these, 55 were allocated to the face-to-face arm, 108 to the online arm, and 23 to the control arm. By the end of the study, dropout rates were 11, 20, and 22% in the three arms, respectively. The number of PLA sessions was adjusted according to participant numbers: six sessions were conducted in the face-to-face arm, eight in the online arm, and none in the control arm. The total costs, including start-up, implementation, and monitoring and evaluation, were £24,158 (INT$36,382) for the face-to-face arm, £44,007 (INT$66,276) for the online arm, and £7,827 (INT$11,787) for the control arm. However, costs incurred in the control arm were excluded from the total programme cost.

We estimated the cost per beneficiary on an intention-to-treat basis, using the allocated number of participants, as most costs, including start-up costs (57%), staff and capital costs during the implementation stage (32%) (Table 1- Panel 2) had already been incurred by that stage. The average cost per mother was estimated to be £439($INT 661) in the face-to face arm and £407($INT 614) in the online arm (Table 2). Excluding the fixed start-up costs, the implementation cost per mother reduced to £208 ($INT 314) for the face-to-face arm and £177($INT 266) for the online arm. This implies that recruiting an additional participant, engaging her in PLA women’s groups, and following up on behaviour change and child health outcomes would require an additional INT$314 in the face-to-face arm and INT$266 in the online arm (Table 2).

**Table 2:** Cost-efficiency indicators.

Trial arm	Amount (2022 £)	Amount (2022 $INT)
Face to face arm:		
Total cost per mother/beneficiary	439	661
Implementation cost per mother/beneficiary	208	314
Online arm:		
Total cost per mother/beneficiary	407	614
Implementation cost per mother/beneficiary	177	266

In Newham, the total cost of NEON delivery was estimated to account for approximately 0.047% of the borough’s expenditure on children’s development during the 2020–21 fiscal year (29). Scaling up the intervention to cover all 10,967 children under the age of 2 in this borough, assuming one caregiver per child, the cost share would rise to roughly 0.491% if implemented face-to-face and 0.418% if implemented online. In Tower Hamlets, the total cost was equivalent to about 0.003% of Tower Hamlets’ spending on children’s development during the same period (30).

### 3.2 Sensitivity analysis

The sensitivity analyses for the total cost per mother are presented in Table 3. The total cost per mother was moderately sensitive to the number of participants (i.e., coverage), increasing by about 10% when calculated for those remaining at mid-PLA sessions (51 in the face-to-face arm and 97 in the online arm) and by about 20% when restricted to the 135 mothers who completed the study (Table 3).

**Table 3:** Sensitivity Analysis on total cost per mother ($INT).

Parameter	Base Case	Lower Bound	Higher Bound
Number of eligible participants	Until first PLA (#55; #108)	Until mid PLA (#51; #97)	Until last PLA (#49; #86)
Face to face arm	661	728 (+ 10%)	790 (+ 20%)
Online arm	614	680 (+ 11%)	750 (+ 22%)
Capital good depreciation rate	20%	10%	30%
Face to face arm	661	649 (− 2%)	671 (+ 2%)
Online arm	614	603 (− 2%)	622 (+ 1%)
Joint cost allocation	Reported	Less to Implementation	More to Implementation
Face to face arm	661	527 (− 20%)	796 (+ 20%)
Online arm	614	474 (− 23%)	754 (+ 23%)

The total cost per mother was largely insensitive to variations in the depreciation rate of capital goods, as capital expenses represented a small proportion of the overall cost. Specifically, as the depreciation rate increased from 10 to 30%, the average cost per mother rose by around 3–4% (Table 3).

Furthermore, the unit cost was notably sensitive to the joint cost allocation. Increasing or decreasing the allocation to implementation activities by 20 percentage points resulted in a corresponding change of just over ±20% in the total cost per mother.

## 4 Discussion

This study aims to assess the costs and affordability of NEON – an early childhood intervention delivered via PLA cycles with women’s groups to improve South Asian infants’ health in London. The intervention cost £68,165 ($INT 102,658) in total, equivalent to less than 0.1% of child development annual spending of local authorities. The average cost per mother was estimated to be £439($INT 661) in the face-to face arm and £407($INT 614) in the online arm, while the marginal cost of recruiting one additional participant, excluding fixed start-up costs, was £208 ($INT 314) for the face-to-face arm and £177($INT 266) for the online arm. The total cost of covering all children under the age of 2 in each borough was estimated at approximately 0.4–0.5% of the borough’s annual child development expenditure.

Precise health outcome measurement, such as DALYs and QALYs, was beyond the scope of this pilot feasibility randomised controlled trial; nevertheless, we observed substantial intermediate behavioural changes. Compared with the control arm, children in the intervention arms showed large increases in food responsiveness (from 29 to 72%) and food enjoyment, alongside reductions in emotional overeating (from 15 to 5%). Parental feeding practices also improved: emotional feeding decreased from 20 to 11% and instrumental feeding from 65 to 38%. Video analyses further identified a reduction in force-feeding behaviours. No significant differences were observed in children’s BMI z-scores, likely attributable to the limited six-month follow-up period (31).

PLA with women’s groups aimed at improving early childhood health has been found to be highly cost-effective across a range of outcomes in resource constrained settings. The cost-effectiveness of a community mobilisation intervention through women’s groups to improve maternal and neonatal care was estimated at $79 per disability-adjusted life year averted in Malawi (MaiKhanda trial) (32), $220 to $393 per year of life lost averted in rural Bangladesh (33), and $14 per cognitive development score gained in rural Viet Nam (38). However, few studies have assessed the cost-effectiveness of PLA with women’s groups in enhancing early childhood nutrition (21, 34, 35). A nutrition-focused intervention targeting children under 2 years of age in Pakistan significantly improved weight-for-height z-scores, cognitive, language, and motor development, but did not provide formal cost-effectiveness estimates (20). Similarly, a community-based strategy in India promoting infant feeding, hygiene, care, and stimulation that demonstrated an improvement in length-for-age z-score was associated with a cost of $302 per woman enrolled (21). No evaluations of costs or cost-effectiveness of PLA with women’s groups aimed at improving early childhood health and development have been conducted in the UK or equivalent high-income settings. To our knowledge, this study is the first to generate evidence in this setting; we hope our findings inform future early childhood nutrition interventions via community-led PLA in both LMICs and high-income countries.

The incremental start-up cost ($INT 58,021), accounting for more than half of the total cost, is expected to be lower for future replicated or scaled-up interventions. Much of the groundwork, including evidence-based implementation strategies (15), staff training, and community partnership establishment, has already been explored and streamlined. Insights, methodologies, and resources from previous projects can facilitate smoother project initiation, reduce trial-and-error phases, and optimise resource allocation, therefore reducing start-up efforts.

Moreover, as the intervention coverage increases, economies of scale may further reduce the unit cost. In community-based women’s groups, higher coverage allows for the distribution of fixed costs, such as those for training, coordination, group facilitation and overheads, across a larger number of participants, thereby lowering the cost per unit of service delivered. A similar intervention conducted in rural India on a larger scale (1,253 women covered) estimated their average cost per mother to be INT$ 302 (21). Though the main drivers of the cost gap are differences in price and cost of living, economies of scale also play a significant part.

However, it should be noted that economies of scale may lose their effectiveness as coverage expands to include the majority of the population. Reaching traditionally underserved and marginalised groups often requires additional effort (36). Those groups typically face greater barriers to accessing the programme due to their socioeconomic status, geographical location, disability, or other factors. Ensuring their inclusion may necessitate extra actions, resources, and strategies beyond those required for more privileged or accessible populations. Research on scaling up nutrition interventions has shown that when coverage expands to 80% of the target population, the unit cost remains relatively stable. However, as the effort shifts to reach the remaining 10%—pushing coverage from 80 to 90%—the unit cost can increase three to four times compared to earlier stages of the scale-up (ibid).

Integrating new interventions into existing services can ease implementation challenges and reduce overall costs. However, maintaining service coverage and quality, and controlling costs, in subsequent replications may prove challenging (8, 18, 19). The NEON intervention, implemented via PLA women’s groups, was founded on a community-led model that integrated into the regular care delivery systems of local communities. This approach allowed the incorporation of the local context, such as the culture, beliefs, and existing practices, leading to boosted accessibility and acceptability (8, 19). Nevertheless, the extensity and quality of service delivery could be largely dependent on the capabilities of the local health system (8). In contexts with limited resources and weaker public health infrastructures, marginalised populations may remain underserved, which could undermine the central goal of promoting equitable early childhood development. Moreover, integrating new interventions into established health systems may impose additional workloads on health workers, potentially affecting their well-being, performance, and the overall quality of care they provide (23). By leveraging existing personnel and public resources, the NEON intervention obviated the need for recruiting specialised staff and procuring relevant resources and reserved potential to be efficiently replicated on a larger scale. However, replications in less resourced contexts may incur additional costs to address these systemic challenges.

Costs are also sensitive to contextual factors, such as local price levels and logistical considerations. For example, staff salaries, which account for 45% of the total cost, could be lower if the interventions were expanded or replicated in regions with lower average salaries than London. Additionally, nearly half of the total cost was allocated to monitoring and evaluation. In this trial, extensive efforts were made to ensure proper implementation and to conduct frequent, detailed assessments at each stage, which contributed significantly to the overall costs. In the future, once the intervention validity is established and a proven implementation system is in place, these monitoring efforts could be scaled back, leading to further cost reductions.

The total cost was also inflated due to the impact of the COVID-19 pandemic. The overall trial duration has been extended to accommodate interruptions caused by lockdowns and sick leaves (31). Stringent infection prevention and control measures have been implemented, such as enhanced cleaning and disinfection protocols, increased use of PPE, and modifications to facility layouts. These situations increased the operational costs. But we expect the increase would not be significant since we carried out remote working and online PLA meetings.

## 5 Conclusion

The delivery of NEON largely falls within local authorities’ budgets for childhood development. The unit cost is sensitive to both the level of coverage and the joint cost allocation across activities. It is anticipated that the unit cost will further decrease as the intervention scales up, with costs being spread across a larger number of participants and monitoring and evaluation efforts being reduced once validated and well-developed implementation systems are established.

## Data Availability

The datasets presented in this article are not readily available because they contain sensitive information that could compromise participant confidentiality. Requests to access the datasets should be directed to Logan Manikam, logan.manikam.10@ucl.ac.uk.

## Group members of the NEON Steering Team collaborators

In addition to the authors, members of the NEON steering team consist of Prof Atul Singhal, Prof Mitch Blair, Dr. Sonia Ahmed, Amelie Gonguet, Gary Wooten, Dr. Ian Warwick, Vaikuntanath Kakarla, Phoebe Kalungi, Prof Richard Watt, Prof Audrey Prost, Dr. Edward Fottrell, Ashlee Teakle, Keri McCrickerd, Dr. Rana Conway, Professor Lisa Dikomitis, Mari Toomse- Smith, Scott Elliot, Julia Thomas, Aeilish Geldenhuys, Chris Gedge, Kristin Bash, Dr. Dianna Smith, Kate Questa, Dr. Megan Blake, Prof Gary Tse, Dr. Queenie Law Pui Sze, Gavin Talbot, Dr. Chiong Yee Keow, Dr. Angela Trude, Prof Lindsay Forbes, Dr. Nazanin Zand, Lakmini Shah and Yeqing Zhang.
